# Efficacy of compatible acupoints and single acupoint versus sham acupuncture for functional dyspepsia: study protocol for a randomized controlled trial

**DOI:** 10.1186/s13063-019-3875-5

**Published:** 2020-01-14

**Authors:** Le Guo, Xin Huang, Li-Juan Ha, Jing-Zhou Zhang, Jia Mi, Ping-Hui Sun, Xi-Ying Han, Ying Wang, Jing-Lin Hu, Fu-Chun Wang, Tie Li

**Affiliations:** 1 Department of Acupuncture and Moxibustion, Changchun University of Chinese Medicine, Changchun, 130117 China. Department of rehabilitation, Changchun hospital of traditional Chinese medicine, Changchun, 130022 China; 2 Jilin Ginseng Academy, Changchun University of Chinese Medicine, Changchun, 130117 China; 3 Department of Acupuncture and Moxibustion, Changchun University of Chinese Medicine, Changchun, 130117 China; 4 Department of Disease Prevention, First Affiliated Hospital to Changchun University of Chinese Medicine, Changchun, 130021 China; 5 Department of Endocrinology, First Affiliated Hospital to Changchun University of Chinese Medicine, Changchun, 130021 China; 6 Department of Epidemiology and Health Statistics, School of Public Health, Jinlin University, Changchun, 130021 China; 7 DDepartment of pharmacy, Changchun University of Chinese Medicine, Changchun, 130021 China

**Keywords:** Acupuncture, Compatible acupoints, Single acupoint, Functional dyspepsia, Randomized controlled trial

## Abstract

**Background:**

Acupoint selection is a key factor in the treatment of diseases and has not been well studied. The aim of this trial is to explore the differences in efficacy between compatible acupoints and a single acupoint for patients with functional dyspepsia (FD).

**Methods:**

This randomized controlled trial will be conducted in the First Affiliated Hospital of Changchun University of Chinese Medicine in China. Two hundred and sixteen FD patients will be randomly assigned to the compatible acupoints group, single acupoint group, or sham acupuncture group. This trial will include a 1-week baseline period, a 4-week treatment period, and a 4-week follow-up period. During the 4-week treatment period, patients will receive 20 sessions of acupuncture (weekly cycles of one session per day for 5 consecutive days followed by a 2-day break). The primary outcome will be a change in the Nepean Dyspepsia Life Quality Index from baseline to after the 4-week treatment period. Secondary outcome measures will include the dyspeptic symptom sum score, Overall Treatment Effect questionnaire, and 36-item Short Form survey. Adverse events also will be recorded. Ultraweak photon emission and metabolomics tests will be performed at baseline and at the end of treatment to explore the mechanisms of the differences between compatible acupoints and a single acupoint.

**Discussion:**

The results of this trial will allow us to compare the difference in efficacy between compatible acupoints and a single acupoint. The findings from this trial will be published in peer-reviewed journals.

**Trial registration:**

Acupuncture-Moxibustion Clinical Trial Registry, AMCTR-IPC-18000176, registered on 4 March 2019; Chinese Clinical Trial Registry, ChiCTR1900023983, registered on 23 June 2019.

## Background

Functional dyspepsia (FD) is a functional gastrointestinal disorder characterized by persistent upper dyspeptic symptoms without organic lesions [1]. According to the Rome IV Standard, FD is divided into two subgroups: epigastric pain syndrome (EPS) and postprandial distress syndrome (PDS) [1]. The prevalence of FD is between 8% and 12% according to a recent cross-sectional study [2]. FD has negative impacts on health and results in a high economic burden [3].

Currently, the treatment for FD mainly includes lifestyle adjustment [4], eradication of *Helicobacter pylori* [5], antacid drugs [6], and prokinetic drugs [7]. However, a standard management for FD has not yet been established, and satisfactory pharmacotherapy is also unavailable [8]. As a result, an increasing number of patients tend to seek complementary and alternative therapies, such as acupuncture [9].

In recent years meta-analyses have suggested that acupuncture could alleviate symptoms of dyspepsia, regulate related negative emotions, and improve the quality of life in patients with FD [10, 11]. However, the number of acupoints selected in these clinical studies has varied. Acupoint selection is a key factor influencing the effect of acupuncture therapy [12]. The aim of this trial was to explore the differences in efficacy between the use of compatible acupoints and a single acupoint for patients with FD.

## Method/design

### Study design

**Fig. 1:**
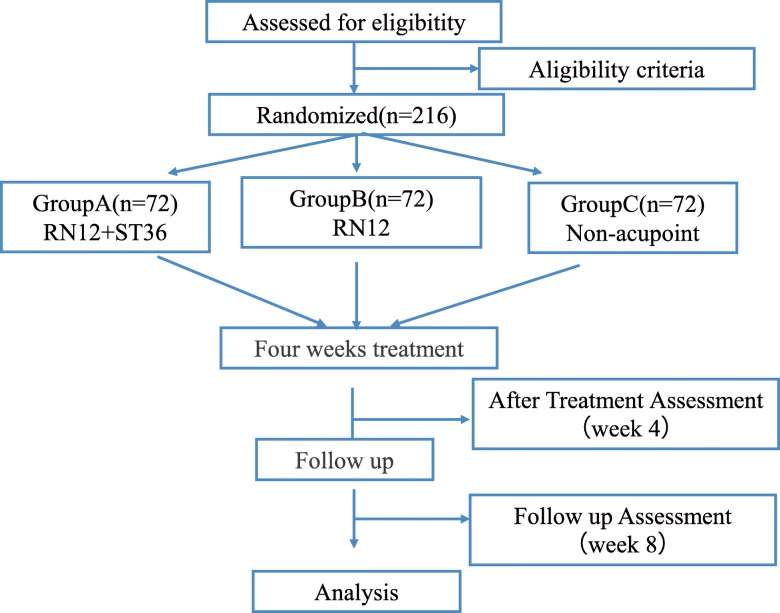
Study flow chart

This is a parallel randomized controlled study conducted in the First Affiliated Hospital of Changchun University of Chinese Medicine in China. Two hundred and sixteen FD patients will be recruited and randomized into one of three groups in a 1:1:1 ratio of the compatible acupoints group, single acupoint group, and sham acupuncture group. The three phases include a 1-week baseline period, a 4-week treatment period, and a 4-week follow-up period. Figure 1 shows the flow diagram of the trial. The study will be conducted in accordance with the Declaration of Helsinki and has been approved by the Ethics Committee of the First Affiliated Hospital of Changchun University of Chinese Medicine (CCZYFYLL2018–011-1). We have registered in the Acupuncture-Moxibustion Clinical Trial Registry (AMCTR-IPC-18000176) and Chinese Clinical Trial Registry (ChiCTR1900023983). Written informed consent will be obtained from each participant before randomization. The Standard Protocol Items: Recommendations for Interventional Trials (SPIRIT) 2013 checklist [13] is provided in Additional file 1.

### Participants

#### Participation criteria

Participant inclusion criteria are as follows:

1. met the diagnostic criteria of Rome IV [1] for functional dyspepsia
2. signed the written informed consent form
3. aged between 18 and 50 years old (either sex)
4. had not taken a drug that may affect dyspepsia in the 15 days prior to the study
5. not participating in any other research

#### Exclusion criteria

Participant exclusion criteria are as follows:

1. dyspepsia symptoms caused by organic disease, metabolic disease, or drugs
2. serious physical or mental diseases, such as cardiovascular diseases, lung diseases liver diseases, kidney diseases, and hematopoietic diseases
3. aggravated malignant tumors or other serious consumptive diseases, infectious diseases, or bleeding diseases
4. fear of acupuncture or infection related to the locations of ZuSanli (ST36) and ZhongWan (RN12)
5. inability to cooperate with gastroscopy, severe anxiety, depression, or fear of dark spaces
6. pregnancy or lactation in women

### Recruitment, randomization, allocation concealment, and blinding

Notices will be published and distributed through advertisements, hospital websites, and notice boards. Research assistants will obtain signed written informed consent forms. Additional consent provisions for the collection and use of biological specimens will be provided to the participants. Participants will be randomly divided into the compatible acupoint group, the single acupoint group, or the sham acupuncture group in a 1:1:1 ratio. The randomization sequence will be generated with a random number table by an independent statistician. A sealed envelope will be used to hide the group assignments. Then, the envelopes will be numbered in sequential order from 1 to 216 and stored by a research assistant who does not take part in enrolling patients. When an eligible patient is enrolled into the study, an envelope will be opened by the research assistants who are responsible for enrolling the patients. The acupuncturists will not be masked due to the nature of acupuncture. However, participants, outcome assessors, and statisticians will be unaware of the group assignments. The participant’s allocated intervention will be not revealed until the statistical analysis is completed.

### Intervention

Participants will be randomly allocated into one of three groups: A, the compatible acupoints group; B, the single acupoint group; or C, the sham acupuncture group (control group). Two acupuncturists, who are from Chinese Medicine Practitioners in China and have at least three years of clinical experience in acupuncture practice, will perform all acupuncture sessions. Several training workshops will be provided prior to the commencement of the study to ensure that the skills of the acupuncturist are consistent. During the 4-week treatment period, patients will receive 20 sessions of acupuncture (4 weekly cycles of one treatment per day for 5 consecutive days, followed by a 2-day break). Sterile needles (0.25 mm × 40 mm/ 0.25 × 25 mm, Hwato, Suzhou, China) will be used in the trial. All acupuncture treatments and laboratory tests will be provided for free to improve adherence to the intervention protocol. Patient and acupuncturist signatures will be required after each acupuncture session to monitor adherence. Other treatments that may affect the dyspepsia symptoms will be prohibited, such as antacids, prokinetics, non-steroidal anti-inflammatory drugs, and antidepressant drugs.

#### Acupuncture groups A and B

**Fig. 2:**
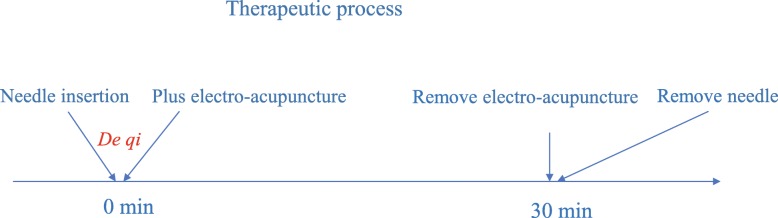
Manipulation procedure.

Acupuncture will be applied at RN12 and bilateral ST36 in group A (compatible acupoints group) but will be applied only at RN12 in group B (single acupoint group). According to our previous reviews, ZuSanli (ST36) and ZhongWan (RN12) are the most frequently used compatible acupoints in gastrointestinal motility disorders [14, 15]. The acupoint locations are based on the “2006 People’s Republic of China National Standard” (GB/T1234–2006), and the acupuncture operation is based on the textbook of the 12th Five-Year Plan of the Ministry of Health. In particular, the skin will be cleaned with alcohol (75%), the sterile needles will be inserted perpendicularly 21–26 mm into the acupoints, and then they will be lifted and thrusted for de qi (a compositional sensation including soreness, numbness, distention, and heaviness). One auxiliary needle will be shallowly pricked in a position 2 mm away from the needle (proximal part). Each needle used for acupoints and their auxiliary needles will be connected to an electroacupuncture apparatus (Hwato SDZ-V Acupoint Nerve Stimulator, Suzhou Medical Co., Ltd.) for 30 min. The frequency will be 60/100 Hz, and the electric current will vary from 0.1 to 1.0 mA until the patients feel comfortable. The manipulation procedure is shown in Fig. 2

#### Control group

The nonacupoint puncture to be used in group C is located in the medial arm on the anterior border of the insertion of the deltoid muscle at the junction of the deltoid and biceps muscles, and this is a location that has been used in several trials [16–18]. Then, the sterile needles will be perpendicularly inserted 21–26 mm into the acupoints but not for de qi. One auxiliary needle will be shallowly pricked in a position 2 mm away from the needle (proximal part). The electroacupuncture apparatus will be the same as the one used for the acupuncture groups.

### Outcomes

**Fig. 3:**
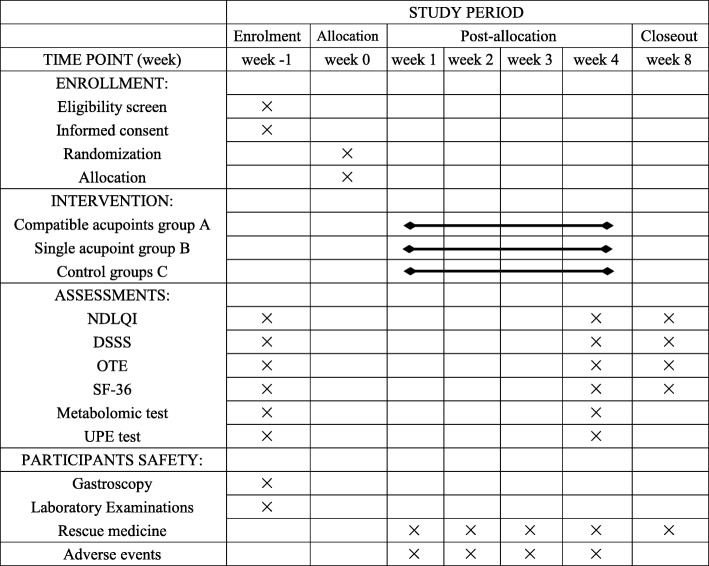
Schedule of enrollment, intervention and assessments for this study protocol. NDLQI: Nepean Dyspepsia Life Quality Index; DSSS: dyspeptic symptom sum score; OTE: overall treatment effect; SF-36: 36-item short form survey; UPE: ultraweak photon emission

The following outcomes will be assessed by independent outcome assessors. These assessors will be trained before participating in this trial and will be blinded to the group assignments. The outcome assessors will contact the participants on the phone up to three times if the participants do not respond to the assessment. The outcome assessments will be performed at three time points: at baseline, at the end of the acupuncture treatments, and at the end of the follow-up period (Fig. 3). Patient privacy information will be separated from the trial data, and the analysis and publication of trial data results will not involve patient privacy information.

#### Primary outcome

The primary evaluation index in the trial will be the change in scores on the Nepean Dyspepsia Life Quality Index (NDLQI) from baseline through the 4-week follow-up period. The NDLQI [19] is a disease-specific scale of quality of life that includes four aspects: life interference (13 items), knowledge/control (seven items), drinking/eating (three items), and sleep/disturbance (two items). Each item contains five options, corresponding to 1 to 5 points. The sum score = 100-[(actual raw score − lowest possible raw score)/raw score range] × 100. Higher scores indicate a better quality of life.

#### Secondary outcomes

##### Dyspeptic symptom sum score (DSSS)

The mean change of dyspeptic symptom sum score (DSSS) [20] from baseline is used to evaluate the severity level of four main symptoms: postprandial fullness, early satiety, epigastric pain, and epigastric burning sensation. Each symptom will be scored as follows: 0 (none), 2 (mild: spontaneous remission within 1 h after meal), 4 (moderate: ease within 1–3 h after meal), and 6 (severe: relief after > 3 h and/or treatment needed). All scores are added up to obtain a sum score between 0 and 24. Higher scores indicate more severe symptoms.

##### Overall treatment effect (OTE)

The mean change in the OTE [21] from the baseline will be evaluated, which is a questionnaire of 0–3 points as follows: 0 points for no relief or exacerbation, 1 point for mild relief, 2 points for obvious relief, and 3 points for complete relief.

##### Health state

The health state will be evaluated by the mean change of SF-36 [22] from baseline, which is a questionnaire consisting of 36 items, including two subscales (physical subscale and psychological subscale). The scores on each subscale range from 0 to 100. Lower scores indicate a worse health state.

#### Laboratory tests

Laboratory tests will be performed at two time points: baseline and at the end of treatment. In the morning, after the participant has fasted for at least 12 h, venous blood and urine will be collected at the First Affiliated Hospital of Changchun University of Chinese Medicine. Metabolomics tests will be conducted at The Jilin Ginseng Academy of Changchun University of Chinese Medicine.

Ultraweak photon emission (UPE) [23, 24] will be carried out with the biological ultraweak photon emission signal acquisition system (PMS09.1) at the Acupuncture and Moxibustion Research Institute of Changchun University of Chinese Medicine. These measurements will be performed at the baseline and at the end of the treatment time points. During the testing period, participants will wear black clothes and enter a darkroom. UPE will be detected at the RN12 acupoint before 12 a.m. over a 20-min period. The purpose of this test is to detect mean changes in UPE before and after acupuncture and to explore the mechanisms of acupuncture treatment on FD based on energy metabolism.

#### Adverse events

Any adverse events reported by the participants will be recorded in the case report form (CRF), including the time, symptoms, signs, degree, duration, laboratory test index, treatment and outcome, follow-up, and follow-up time. Common treatment-related adverse events include subcutaneous hematoma, continuous post-needling pain, itching at the sites of the needle insertion, and dizziness.

### Sample size

Based on our clinical experience, changes in the NDLQI scores in the compatible acupoint group, single acupoint group and sham acupuncture group at the 4th week are expected to be 12, 8, and 4, respectively. The standard deviations in the three groups are expected to be 10, 13, and 13, respectively. The two-sided significance level is 0.05.

$$ n=\frac{\psi^2\left(\sum {S}_i^2/k\right)}{\sum {\left({\overline{X}}_i-\overline{X}\right)}^2/\left(k-1\right)} $$

A sample size of 60 patients in each group is estimated to have 80% power to detect significant differences among the three groups. To compensate for a 20% loss to follow-up, the sample size was increased to 72 patients.

### Statistical analysis

The intention-to-treat analysis will be used for all allocated participants in the baseline condition. Baseline characteristics will be summarized across the treatment groups. Missing values will be imputed by the last observation carried forward (LOCF) method. Depending on whether the data are normally distributed, the continuous variables will be described as the mean (standard deviation) or the median (interquartile range). One-way analysis of variance (ANOVA) will be used for comparisons among the three groups. Categorical variables will be described using frequency (percentage) and compared using Fisher’s exact test. The statistical analyses will be performed using SAS 9.1.3. *P* < 0.05 will be considered statistically significant.

All efficacy analyses, which will include all participants who are randomized, will be performed using the intent-to-treat analysis. Primary comparisons (change in the NDLQI scores at week 4 relative to the baseline scores among the three groups) will be made with ANOVA. Pairwise comparisons will use the Student-Newman-Keuls method.

For the secondary outcomes, continuous variables, including the DSSS and SF-36 scores, metabolomics, and UPE, will be compared among the three groups at all follow-up time points using ANOVA. The Student-Newman-Keuls method will be used for pairwise comparisons. The OTE and adverse events will be summarized for each group and compared using Fisher’s exact tests.

Analysis of covariance (ANCOVA) or logistic regression analysis will be used in the sensitivity analysis. The sensitivity analysis will also be performed using a per-protocol analysis, which includes only those who complete ≥16 sessions and have no major protocol violations (e.g., taking other drugs during the trial).

### Monitoring

This trial does not have a Data and Safety Monitoring Committee. The Ethics Committee of the First Affiliated Hospital of Changchun University of Chinese Medicine will be responsible for the safety monitoring.

### Quality control

All researchers will be required to undergo special training that will address the trial design, participant inclusion and exclusion criteria, and proper completion of the paper CRF. The paper CRF will be kept for at least 5 years. The original data will be double-entered into the Epidata system. The private information of patients—including name, telephone number, and ID number—will be anonymous to ensure participant confidentiality. All practitioners who have majored in acupuncture and received an acupuncture degree are qualified doctors of Traditional Chinese Medicine. No allowance is available to promote retention and to complete the patient follow-up. If participants do not respond to the assessment, outcome assessors will contact the participant by phone up to three times. The reasons for loss to follow-up will be fully recorded. The ethics committee will conduct an audit of this trial annually.

## Discussion

According to recent systematic reviews [10, 11, 25], acupuncture is effective in the treatment of FD. However, many factors influence the effects of acupuncture [12], including acupuncture location, stimulation mode, treatment timing, retention time and treatment frequency. Among these factors, acupoint selection is an important factor. We designed a randomized controlled clinical trial to determine the differences between compatible acupoints and single acupoints in treating patients with FD.

Each acupoint has its own characteristics, i.e., its acupoint nature, which refers to the differences in the specificity reflected in the therapeutic effect and also the inherent biological properties of the acupoint. Acupoint nature is affected by many factors, such as the meridians to which the acupoint belongs and the position of the acupoints. Acupoint nature includes universality and specificity. Fully recognizing the universality and specificity of acupoints will be helpful in improving the effect of acupuncture therapy in clinical practice. Previous laboratory studies have suggested that different underlying mechanisms exist between acupuncture at a single acupoint and acupuncture at compatible acupoints in regulating gastric motility in diabetic rats with gastroparesis [26]. Acupuncture at ST36 can promote gastric motility by increasing vagal nerve activation and inhibiting sympathetic nerve activity. Acupuncture at RN12 or at RN12 and ST36 can simultaneously suppress gastric motility by reducing vagal nerve activation and inhibiting sympathetic nerve activity. Li Yuqing and colleagues [27] also found that acupuncture at abdominal acupoints could inhibit gastrointestinal motility in rats by electrogastrogram and other measurement methods.

Acupoint selection is one of the key factors affecting the therapeutic effects of acupuncture. First, acupuncturists should become acquainted with the acupoint nature and fully recognize the universality and specificity of the acupoints under the guidance of traditional Chinese medicine theory. Second, acupuncturists should follow the suitable selection principles and methods. The compatibility of different acupoints with similar therapeutic effects can synergize the effects of acupuncture to achieve the goals of clinical treatment.

To date, however, acupoint selection has not been uniform for the treatment of FD. Zeng Fang and colleagues [28] selected ST34, ST36, ST40, and ST42. Yang Jingwen and colleagues [29] chose DU20, RN12, ST25, RN6, PC6, RN17, ST36, and SP4. According to reviews of the literature, ST36 and RN12 are the most frequently used compatible acupoints in gastrointestinal motility disorders [14, 15]. Therefore, ST36 and RN12 were selected for this trial. A clinical trial has suggested that the effects of compatible acupoints on the main symptom index of gastric palsy and the 180-min gastric residual rate were better than those of a single acupoint [30].

This study also has some limitations. First, the design is a single blind trial. When participants sign the informed consent form, they are informed that this project is to study the efficacy of three acupuncture methods, and patients will be randomly assigned to any one of the groups. Due to the nature of acupuncture, the masking of acupuncturists is quite difficult to achieve. However, the statistician will be masked. Second, this is a single-center trial in China, and whether the results will have generalized significance for other patients is unknown. At the end of this trial, we hope that the results will provide more reliable evidence for clinical acupoint selection in the treatment of FD.

## Trial status

Protocol version V1.1 dated 10 May 2018. The first participant was enrolled on 11 April 2019. Recruitment will be complete on 10 May 2022.

## Supplementary information

**Additional file 1.** Checklist of the Standard Protocol Items: Recommendations for Interventional Trials (SPIRIT) guidelines.

## Data Availability

All data collected during the trial will be available from the corresponding author (Tie Li, litie1999@126.com) for anyone who wishes to access the data immediately following publication.

## Abbreviations

CRF: Case report form
DSSS: Dyspeptic symptom sum score
EPS: Epigastric pain syndrome
FD: Functional dyspepsia
NDLQI: Nepean Dyspepsia Life Quality Index
OTE: Overall treatment effect
PDS: Postprandial distress syndrome
RCT: Randomized controlled trial
SF-36: 36-item short form survey
TCM: Traditional Chinese medicine
UPE: Ultraweak photon emission
